# Analyzing Dyadic Sequence Data—Research Questions and Implied Statistical Models

**DOI:** 10.3389/fpsyg.2017.00429

**Published:** 2017-04-11

**Authors:** Peter Fuchs, Fridtjof W. Nussbeck, Nathalie Meuwly, Guy Bodenmann

**Affiliations:** ^1^Department of Psychology, Bielefeld UniversityBielefeld, Germany; ^2^Department of Psychology, University of FribourgFribourg, Switzerland; ^3^Department of Psychology, University of ZurichZurich, Switzerland

**Keywords:** observational data, dyadic interaction, relationship research, dyadic data analysis, behavioral interactions, sequence data, interval sampling, DySeq

## Abstract

The analysis of observational data is often seen as a key approach to understanding dynamics in romantic relationships but also in dyadic systems in general. Statistical models for the analysis of dyadic observational data are not commonly known or applied. In this contribution, selected approaches to dyadic sequence data will be presented with a focus on models that can be applied when sample sizes are of medium size (*N* = 100 couples or less). Each of the statistical models is motivated by an underlying potential research question, the most important model results are presented and linked to the research question. The following research questions and models are compared with respect to their applicability using a hands on approach: (I) Is there an association between a particular behavior by one and the reaction by the other partner? (Pearson Correlation); (II) Does the behavior of one member trigger an immediate reaction by the other? (aggregated logit models; multi-level approach; basic Markov model); (III) Is there an underlying dyadic process, which might account for the observed behavior? (hidden Markov model); and (IV) Are there latent groups of dyads, which might account for observing different reaction patterns? (mixture Markov; optimal matching). Finally, recommendations for researchers to choose among the different models, issues of data handling, and advises to apply the statistical models in empirical research properly are given (e.g., in a new r-package “DySeq”).

## Introduction

The primary purpose of this contribution is to give an overview of statistical models that allow for analyzing behavioral coding of dyadic interactions. To this end, specific research questions that often arise in the analysis of dyadic interactions are linked to the corresponding statistical models. Furthermore, it will be shown how to estimate these models, and how to interpret these models relying on an empirical example. Hence, we aim at promoting the presented analyses and to make them more accessible for applied researchers, especially in the field of relationship research. Therefore, a commented R-script, example data and an R-Package with additional functions will be provided.

In psychology, especially in relationship research, many research questions concern dynamics of social interactions. The smallest unit, in which interactions can occur, is a *dyad*. In principle, dyads can be categorized by their *type, linkage*, and *distinguishability* (Kenny et al., 2006). The specification of the *type of dyad* depends on the roles of the two dyad members (e.g., mother-child dyads, heterosexual, or homosexual couples). *Linkage* describes the mechanisms by which (or the reason why) the two dyad members are linked. The two partners may be voluntarily linked (e.g., two friends), they may be linked by kinship (e.g., mother and child), there may be an experimental linkage (e.g., the two partners, who do not know each other before the experiment, are asked to solve an experimental task together), or there may be a yoked linkage, that is, the two members of the dyad experience the same environmental influences, but they do not interact with each other (see Kenny et al., 2006).

Finally, dyads can be considered distinguishable or indistinguishable. In distinguishable dyads, there is at least one relevant quality (role) of the two members, which allows for a clear distinction between the two (e.g., mothers and daughters). In indistinguishable dyads, there is no such quality (role) that may differentiate between the two members (e.g., monozygotic twins). The choice of the statistical model for the analysis of dyadic data strongly depends on the distinguishability of the two partners (for an overview see Kenny et al., 2006). In this contribution, we focus on distinguishable dyads (e.g., heterosexual couples).

As in many other fields of psychology, research on dyadic interactions relies mainly on self- and partner reports. Typically, these reports describe an overall evaluation of a psychological mechanism (e.g., evaluation of the joint efforts to cope with stress) or they describe typical patterns of behaviors, which the two members of the dyad experience when being together (e.g., how they jointly deal with the stress of one partner). However, self-and other reports potentially suffer from different biases: Self- (and partner) reports about behavior may integrate an evaluative perspective about past behaviors but also social comparisons with other couples which may be top-down biased by overarching constructs as relationship satisfaction, for example. They may also be biased due to self-deception, exaggeration, social desirability, mood dependency, or oblivion (e.g., Lucas and Baird, 2006).

Hence, in many contributions authors call for multimethod measurements (e.g., Eid and Diener, 2006) including behavioral coding and the analysis of behavioral interactions. However, the analysis of behavioral interactions requires statistical approaches that are not commonly used in psychology. With this contribution, we aim at informing researchers about how to analyze dyadic observational data using prototypical research questions. We will focus on sequentially coded data (*interval sampling*) as this is the preferred method when different behaviors can be observed in interaction sequences (Kenny et al., 2006). Interval sampling implies that the observation period is divided into time intervals of the same length (e.g., 8 min may be divided into 48 intervals of 10 s length each). For each interval, it is coded if a particular behavior occurred (0 = no; 1 = yes). The resulting entries in the data matrix are sequences of so-called states describing the (non-) occurrence and re-occurrence of that particular behavior. There are as many sequences for an observational unit (e.g., a couple) as there are behaviors of interest (e.g., one sequence for the behavior of the 1st partner and one sequence for the behavior of the 2nd partner).

**Figure 1:**
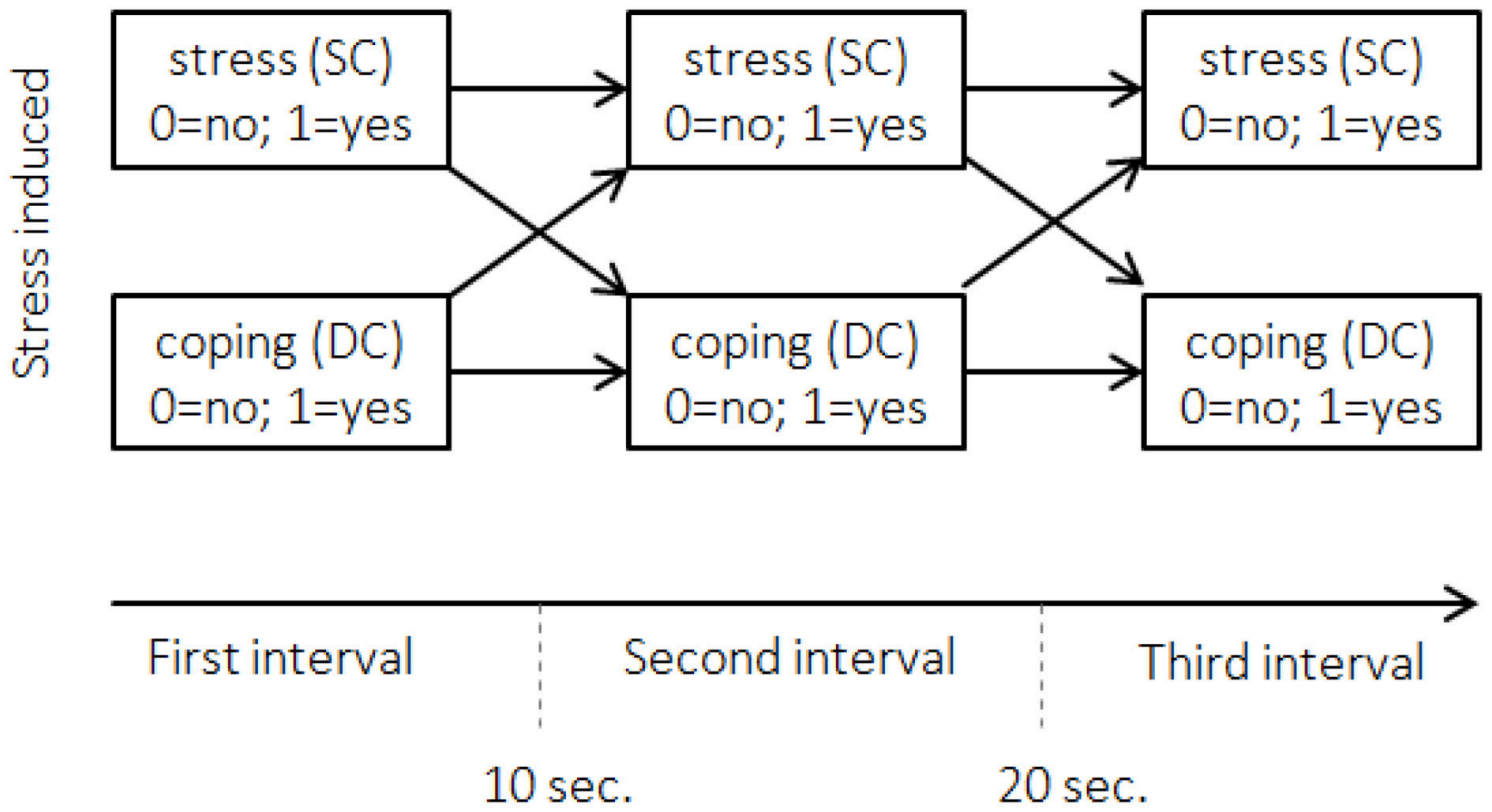
**Interval sampling for the example dataset**. Displayed are the first three 10-s intervals after stress was induced. Horizontal arrows between the boxes refer to actor effects. Crossed arrows refer to partner effects. Stress (SC): did stress communication occur? Coping (DC): did dyadic coping occur?

Figure 1 depicts an example of interval sampling for two behaviors: Stress communication by one partner (SC) and coping reaction by the other partner (e.g., dyadic coping: DC). Hence, the dyads form the observational units producing two interdependent sequences. The entries in the data matrix indicate if the behavior occurred within a given sequence (interval), 1-1-0 for stress communication, for example, indicates that the 1st partner communicated her or his stress in the first interval, did so in the 2nd but did not communicate her or his stress in the 3rd interval.

## Conceptual models for dyadic interactions

**Figure 2:**
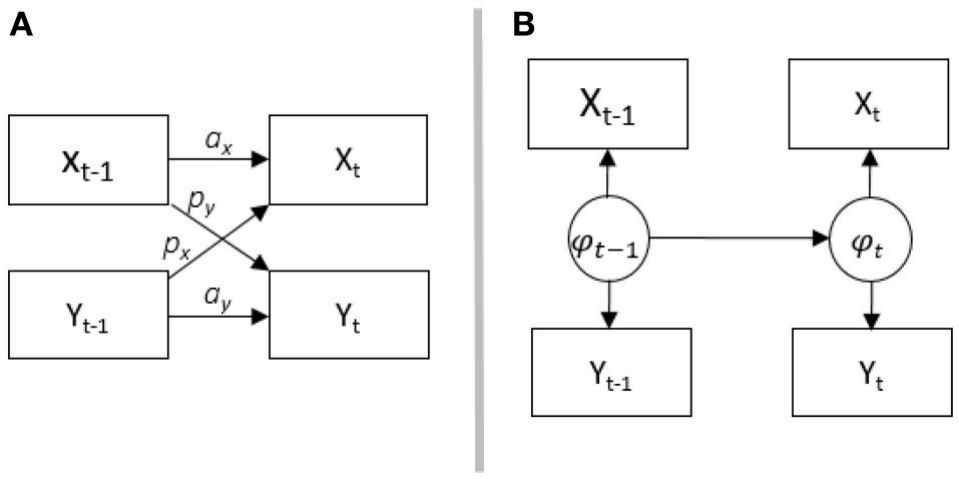
**(A)** shows the conceptuell Actor-Partner-Interdependence Modell (APIM), a_x_ is the actor effect for variable x, a_y_ is the actor effect for variable y, p_x_ is the partner effect for variable x, p_y_ y is the partner effect for variable y; **(B)** shows the common fate model. Both partners are influenced by the latent common variable *phi*. The APIM is adapted from Cook and Kenny (2005).

In order to understand associations in dyadic data, three conceptual models have been introduced (see Kenny, 1996): The *Actor-Partner Interdependence Model* (APIM), *mutual influence*, and the *common fate model*. Although these three models were originally designed for scaled (metric) cross-sectional data, there are extensions and applications to longitudinal and sequential data for the APIM in Kenny et al. (2006). A longitudinal adaption for the common fate model was presented by Ledermann and Macho (2014), yet to our knowledge no adaptations of the common fate or mutual influence model for sequence data exist. Figure 2 shows the adaptation of the APIM (A) and our analogous adaptation of the common fate model (B) for sequence data.

In the standard (cross-sectional) form, the *APIM* is used for modeling influences within (actor effect) and across (partner effect) partners. To this end, path analysis can be used to model two regressions simultaneously. Considering heterosexual couples, for example, one could be interested in knowing if coping competencies affect relationship satisfaction. The male partner's relationship satisfaction may depend on his own coping competencies (actor effect) but also on his partner's (her) competencies (partner effect). The same is true for female relationship satisfaction which may depend on her partner's (his) coping competencies (partner effect) as well as her competencies (actor effect).

The APIM has been adapted for the analysis of longitudinal metric data (Cook and Kenny, 2005) and sequence data (Kenny et al., 2006). In the adapted version for metric data (see also Figure 2A), the same constructs are repeatedly measured over time (e.g., her SC and his DC). Effects between two time intervals within one partner (female partners' SC at time *t*–1 to SC at time *t*) are called *actor effects*, which correspond to autoregressive effects in time-series analysis. Effects from one partner at *t*–1 to the other partner at *t*, are called *partner effects* (female partners' SC at time *t*–1 to male partners' DC at time *t*). These effects correspond to cross-lagged effects in time-series analysis. The adaptation of the APIM for binary categorical sequence data is depicted in Figure 1. In this adaptation, the occurrence of male partners' behavior (here DC) at time *t* is predicted by their immediate previous behavior at *t*–1 (*men's actor effect*), and by the behavior of their partners at *t*–1 (*men's partner effect*). The occurrence of female partners' behavior (here SC) at time *t* is predicted by their behavior at *t*–1 (*women's actor effect*), and by the behavior of their partners at *t*–1 (*women's partner effect*).

In the *common fate model*, it is assumed that there is a property of the couple which influences both partners' behaviors. Consider a conflict between partners, where the conflict describes the couple as a whole and will likely lead to stress communication. In the same vein, female partners' stress may be seen as a property of the couple. Her stress will likely lead to stress communication by her and coping reactions by him. In sequential data and according to the common fate model, her stress communication and his coping reaction may be indicators of a latent status (female partners' stress) which can change over time (see Figure 2B). Hence, changes in the two behaviors are modeled as indicators of one latent variable. Depending on the research question and the underlying assumptions, researchers may choose between the models. In the remainder of this contribution, we will outline possible adaptations, applicability and interpretation of the different models relying on prototypical research questions.

## General research questions

Research questions that arise in relationship research are oftentimes comparable to the following four questions: (1) Is there an association between behaviors of dyad members (e.g., is more frequent SC related to more frequent DC)? (2) And if so, how do the partners interact? Does the behavior of one member trigger an immediate reaction by the other (does SC by one partner evoke a prompt DC reaction by the other)? (3) Or is there an underlying dyadic process, which might account for the observed behavior (is the stress of one partner simultaneously influencing both partners' behavior such that one partner shows SC and the other DC behaviors)? (4) Are the mechanisms producing the behavioral patterns the same for all couples or is there unobserved heterogeneity such that there are different typical response patterns (does the experience of stress lead to very prompt and adequate SC and DC behaviors in all couples resulting in a quick solution to the problem or are there couples struggling with the stressor for a long time)?

## Example data

We will exemplify the typical data structure and illustrate the statistical models throughout this contribution using a sample study from relationship research (Bodenmann et al., 2015). In this sample 198 heterosexual couples living in Switzerland participated. The couples had to have been in the current romantic relationship for at least a year and to use the German language as their primary language. 56% of the women and 40% of the men were students, and their age ranged from 20 to 45 years. During the study, either the woman, the man, or both partners were stressed using the Trier Social Stress Test (TSST; Kirschbaum et al., 1993).

Directly after the stress induction, both partners joint again, and the couple was left alone for 8 min without any further instruction. During this period, which was introduced as time the experimenters would need for some adjustment of the experimental installations (a “fake” waiting-condition), the two partners were filmed. In fact, these 8 min of the waiting situation were at the core interest of the study as this situation was supposed to reveal how partners interact after one or both of them has been stressed. In the remainder, this waiting condition will be called the interaction sequence.

For the sake of simplicity, we consider those 64 couples where only the female partner was stressed and restrict our analyses to her stress communication (sequence 1) and the male partner's support reaction (sequence 2; supportive dyadic coping). Stress communication (SC) includes all verbal and non-verbal behaviors signaling stress. Supportive dyadic coping (DC) includes all verbal and non-verbal behaviors aiming to support the partner's coping efforts. Both behaviors were coded relying on the SEDC (Bodenmann, 1995) in 48 intervals of 10 s length. The data structure hence consists of two interdependent sequences with 48 entries. The presented statistical models can be applied to any comparable data situation.

### Graphical inspection

**Figure 3:**
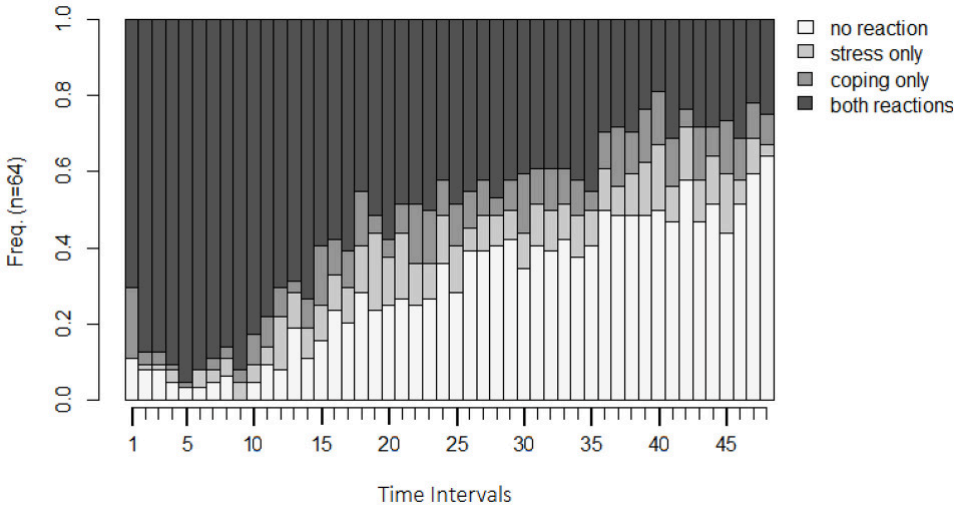
**State distribution plot of the example data**. Y-axis: relative frequency of shown behavior; X-axis: observation intervals; SC only: only stress communication was shown without dyadic coping; DC only: only dyadic coping was shown but no stress communication; none: neither stress communication nor DC were shown; SC+DC: stress communication and dyadic coping were shown.

Before running analyses on the sequential data to answer particular research questions, the inspection of the state distribution plot (Figure 3) allows for a first graphical examination of the paired sequences. To this end, the two sequences have been joined via the state expand procedure (Vermunt, 1993). For each time interval, the joint occurrence/non-occurrence of the two behaviors (see Table 1) is depicted resulting in four possible states per time interval.

**Table 1:** **Resulting four states of the state-expand procedure**.

		**DC (dyadic coping)**	
		**No**	**Yes**
SC (stress communication)	No	None	DC only
	Yes	SC only	SC+DC

*Yes: behavior was shown, no: behavior was not shown*.

**Figure 4:**
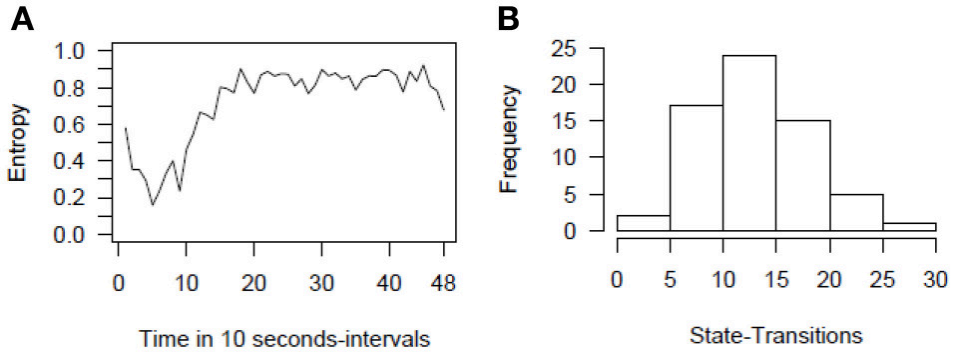
**Entropy plot (A)** and histogram of state-transitions **(B)** for the example data. Entropy refers to the Shannon entropy.

For the sample data, we see that in the beginning, the simultaneous display of stress communication (SC) by women and DC reaction by men (DC) (both reactions) is the most frequently displayed behavior (almost 100%). After roughly 10 intervals (1 min and 40 s), frequencies for the combination of no SC and no DC reaction (no reaction), a stress communication but no DC reaction (SC only), and no SC but a DC reaction (DC only) increase. In the following minutes, the frequencies of SC only and DC only remain rather stable, but frequencies for no reaction increase further while frequencies for both reactions decrease. One possible explanation could be that couples intensively discuss the stressful event in the beginning, and thereon some of the couples manage to be less stressed. Hence, no SC nor DC reaction is necessary for them, while other couples still discuss the stressful event until the end of the interaction sequence. These two mechanisms are also reflected by the Shannon entropy (Shannon, 2001). This coefficient as a measure of dispersion (Figure 4A) shows that couples show very homogeneous behavioral patterns in the first sequences (simultaneous display of SC and DC) and that the couples become more dissimilar at sequence 20. That is, they show all different combinations of SC and DC behavior.

An additional first insight can be gained by inspecting the number of state-transitions as a measure of stability. The number of state-transitions depicts how often couples change from one state into another. A high number indicates frequent changes in a couple's behavior. Hence, the number of state-transitions allows differentiating between volatile couples who frequently change their behavior (high scores) from those who tend not to change their behaviors frequently (low scores). The histogram in Figure 4B shows that the majority of couples change their behavior about 10 to 20 times out of 47 possible changes during the 48 intervals, which is roughly about one to two times within a minute.

**Research Question 1:** Is there an association between behaviors of dyad members (e.g., is more frequent SC related to more frequent DC)? Or, more specifically, do men show DC behavior more frequently if their partners communicate their stress (SC) more frequently?

This very general research question can be answered by calculating the Pearson correlation between the frequencies of the behavior of interest shown by the first partner and the frequencies of the interesting response by the other partner.

For the sample data set, we find a very high Pearson correlation of *r* = 0.86 (*p* < 0.001) between the frequencies of stress communication and DC responses. Which, overall, implies that, in couples with women showing high rates of SC, men tend to show more DC reactions. And vice versa, if women show low rates of SC, men show low rates of DC as well. However, due to the aggregation of the data, information about the direction and the contingency (i.e., promptness) of this association are lost. Hence, it remains an open question if SC leads to prompt DC reactions, if DC leads to prompt SC reactions, or if the association is bidirectional.

**Research Question 2:** Does the behavior of one member trigger an immediate reaction by the other? Or, more specifically, does SC by one partner evoke a prompt DC reaction by the other and vice versa?

Three strategies, which can be used for addressing this research question, will be shown: (A) The *aggregated logit models* approach by Bakeman and Gottman (1997), (B) *Multi-Level logistic regression*, and (C) *Basic Markov Models*.

**Figure 5:**
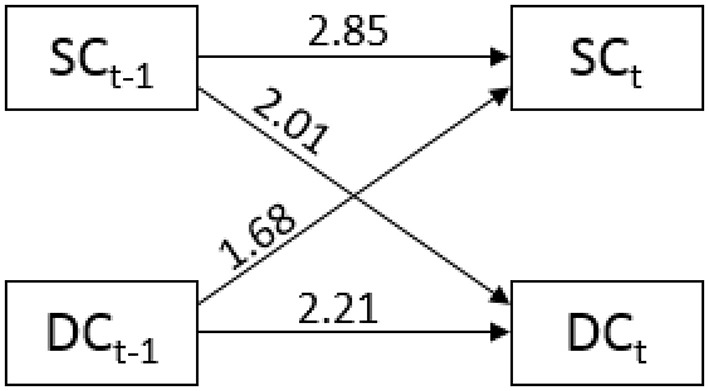
**Odds ratios for partner and actor effects (horizontal arrows) and partner effects (crossed arrows) between stress communication (SC) and dyadic coping (DC)**.

- Bakeman and Gottman's (1997) approach originally consists of three steps, but a fourth step can be added to estimate a full APIM. The first *step* is to produce a state-transition table for each couple. Table 2 shows the transition table for couple 129 as an example. The behavior of interest (DC by men) in interval *t*, is mapped against the combination of the observed behaviors (SC by women and DC by men) in the previous interval *t–*1. Hence, for the data set, 64 tables (one for each couple) are obtained.
  In the second *step*, 64 logit models are estimated (separately analyzing the 64 frequency tables for the 64 couples). The odds of showing the behavior of interest against not showing the behavior are predicted by the past behaviors of the two partners. The first line of Equation (1) shows that these odds are predicted by SC of the women in the preceding interval (*t*–1; *partner effect*), the DC behavior by the men (*t–*1; *actor effect*), and their interaction (SC (t–1)*DC(t–1)). The latter means that the effect of SC at *t–*1 on DC might depend on whether DC was also present at *t–*1 and vice versa.
  (1) $$\begin{array}{l} \frac{P\left(DC_{t}\right)}{1-P\left(DC_{t}\right)}=\text{exp}(\beta_{0})*\exp{\left(\beta_{1}\right)}^{SC_{t-1}}*\\ \exp{\left(\beta_{2}\right)}^{DC_{t-1}}*\text{exp}{\left(\beta_{3}\right)}^{SC_{t-1}*DC_{t-1}}\\ \Leftrightarrow \;ln\left(\frac{P\left(DC_{t}\right)}{1-P\left(DC_{t}\right)}\right)=\beta_{0}+\beta_{1}*SC_{t-1}\\ +\beta_{2}*DC_{t-1}+\beta_{3}*SC_{t-1}*DC_{t-1} \end{array}$$
  Any coding for occurrence and non-occurrence of behavior can be chosen. However, effect coding (1 = behavior is shown and −1 = the behavior is not shown) benefits the most straightforward interpretation of coefficients: exp(β_0_) are the average odds of showing DC against not showing DC; all other exp(β) represent odds ratios (e.g., the factor to which the odds change if SC was present at *t*–1). An exp(β_2_) = 2, for example, would result in two times higher odds for showing DC in the current interval if DC was shown in the previous interval (*t*–1). Whereas, if DC was not shown in the previous interval, the odds would be divided by 2 due the effect coding. The interaction term equals 1, if the two behaviors were shown at the previous interval (*t*–1) but also if they were not shown. Hence, in both cases the odds are multiplied by exp(β_3_). However, if only one behavior was shown (SC = −1 and DC = 1 or SC = 1 and DC = –1) the odds are divided by exp(β_3_).
  The second line of Equation (1) presents the same model in the logit-parameterization: The natural logarithm is taken of both sides of the equation. The parameters are now symmetrically distributed around 0 (no effect) and range from −∞ (negative effect) to −∞ (positive effect). Predicted logits and odds of observing DC can be used for single case analysis as shown for couple 129 in Table 3.
  The *third step* uses the logit parameterization of all models and aggregates the β-parameters by averaging. The β-parameters are *t*-distributed, which allows for standard hypothesis testing. The aggregated βs can be transformed back into odds ratios afterward by applying the exponential function. Table 4 depicts the aggregated estimates for the 64 couples of the sample data. Overall, the odds ratios for intercept, the partner effect, and the actor effect are positive and statistically significant. Therefore, it is principally more likely to observe DC than no DC and the probability increases with preceding stress-related behaviors (either SC and/or DC), yet there is no statistically significant interaction effect.
  The *fourth step* is to enter the second behavior (SC) as the dependent variable and run steps 1 through 3 again. For the sample data, this results in a second aggregated logistic model with women's SC as the dependent variable. Table 5 (first column; SC) shows the estimates for this step. The model shows that a preceding SC increases the probability for showing SC again (*actor effect*) and that preceding DC also increases the probability for SC (*partner effect*).
  Together the third and the fourth step correspond to an APIM, which can be displayed as a path-diagram (see Figure 5). However, this representation is only recommended if interaction effects are small and not significant because the interaction effects cannot be easily integrated into the graphical presentation. If interaction terms are present, a tabulation of the results is more accessible.
- Instead of running multiple logit models, a single multi-level model can be used. The multi-level model has to address three challenges: The longitudinal aspect of sequence data, the fact that sequences are categorical, and the dyadic data structure.
  The longitudinal aspect can be addressed by the multi-level modeling approach itself because it accounts for dependencies within nested observation. Repeated measures, for example, can be seen as multiple observations that are nested within individuals. For sequence data, each but the first interval can be seen as providing an observation that is depending on prior observations. Thus, the example data set provides 94 observations (two variables times 47 intervals) for each dyad.
  Multi-level models can be extended to generalized multi-level models (Hox et al., 2010) allowing for categorical dependent variables (*multi-level logistic regression*). The dependent variable for each interval is the occurrence of the behavior at time interval *t* (occurrence = 0; non-occurrence = 1). The independent variables are the occurrence of the same behavior at *t*-1 (*actor effect*; AE = 1; non-occurrence: AE = −1), and whether the partner showed the other behavior at *t*–1 (*partner effect*; PE = 1) or not (PE = −1).
  The dyadic data structure can be addressed by incorporating a dummy coded moderator variable (e.g., sex), which distinguishes between the two members of a dyad. Equations (2, 4) present the corresponding model equations in logit parameterization:
  Level-1-Equation:
  (2) $$\begin{array}{l} ln\left(\frac{P(DV=1)}{1-P(DV=1)}\right)=\beta_{0j}+\beta_{1j}*AE+\beta_{2j}*PE\\ \;+\beta_{3j}*AE*PE+\beta_{4j}*Sex\\ \;+\beta_{5j}*AE*Sex\\ \;+\beta_{6j}*PE*Sex\\ \;+\beta_{7j}*AE*PE*Sex \end{array}$$
  Conditional Level-1 Equations:
  (3) $$\begin{array}{l} If\;Sex=0:\;ln\left(\frac{P(DV=1)}{1-P(DV=1)}\right)\\ \;=\beta_{0j}+\beta_{1j}*AE+\beta_{2j}*PE+\beta_{3j}*AE*PE \end{array}$$
  $$\begin{array}{l} If\;Sex=1:\;ln\left(\frac{P(DV=1)}{1-P(DV=1)}\right)\\ \;=\left(\beta_{0j}+\beta_{4j}\right)+\left(\beta_{1j}+\beta_{5j}\right)*AE+\left(\beta_{2j}+\beta_{6j}\right)*PE\\ \;+\left(\beta_{3j}+\beta_{7j}\right)*AE*PE \end{array}$$
  Level-2-Equations:
  (4) $$\beta_{0j}=\gamma_{0}+e_{0j};\beta_{1j}=\gamma_{1}+e_{1j};...;\beta_{7j}=\gamma_{7}+e_{7j}$$
  In Equations (2–4), the dependent variable (DV) changes from male to female partners. If male behavior is to be predicted, DV represents DC. If female behavior is to be predicted DV represent SC. In the same vein, AE represents male DC and PE represents female SC at the previous interval if the occurrence of male DC is predicted; AE represents female SC and PE male DC at the previous interval if the occurrence of female SC is predicted. Equation (3) shows the conditional equations for men and women if the variable sex = 0 (upper conditional equation for male partners) or if sex = 1 (lower equation for female partners). Hence, predicting DC behavior by male partners, the upper equation has to be interpreted, predicting female SC, the lower equation has to be interpreted.
  The partner with sex = 0 (male partners) form the reference category. The corresponding average logit, actor, partner, and interaction effects can be obtained directly from the equation. For the non-reference category (sex = 1; female partners), the average logit, actor, partner, and interaction effects differ from the ones for the reference category by the logit parameters associated with the variable sex. For example, β_1*j*_ is the actor effect for the reference category (male partners), and β_5*j*_ shows if the actor effect for the non-reference group (female partners) is larger (positive value) or smaller (negative value); the actor effect for female partners is, hence, depicted by (β_1*j*_ and β_5*j*_). The same is true for partner effects (β_2*j*_ and β_6*j*_) and the actor^*^partner interaction (β_3*j*_ and β_7*j*_) considering female partners. The intercept (β_0*j*_) represents the average logit for the reference group (male partners), and the main effect of sex (β_4*j*_) shows the difference of female partners' intercept from the male partners' intercept.
  Finally, each β_*j*_ is located at level-1 indicating that the regression coefficients may differ between couples. The level-2-equations show that each couple's regression parameters β_*j*_ depend on the average effect over all couples (γ; *fixed effects*) and a couple-specific residual (*e*_*j*_; *random effect*). The fixed effects at level-2 represent the average effects across all couples and conceptually correspond to the average parameters of the aggregated means approach. Additionally, the variances of the random effects indicate how much the regression effects differ between the couples. For example, the variance of the level-2 random component associated to the intercept (*var*(*e*_0*j*_)) describes differences in the mean logits (for the reference group) between couples.
  Moreover, correlations between random effects can be investigated. For example, a positive *cor*(*e*_1*j*_, *e*_2*j*_) indicates that larger (couple specific) *actor effects* are associated with larger *partner effects* in the reference group. Or another example, *cor*(*e*_5*j*_, *e*_6*j*_) is the correlation of the level-2 random effects associated with the differences of the female regression parameters compared to the corresponding male regression parameters. And a positive estimate of *cor*(*e*_5*j*_, *e*_6*j*_) indicates that in couples with larger than average differences for female partners' actor effects we also find larger than average differences for the partner effects. That is couples with higher female actor effects tend to produce higher female partner effects and vice versa.
  Models with different subsets of random effects can be tested against each other for finding a parsimonious model (Hox et al., 2010). For the example data the comparative fit index BIC (Schwarz, 1978) was used. In the best fitting model we only specified a random intercept *var*(*e*_0*j*_), random actor *var*(*e*_1*j*_) and random partner effects *var*(*e*_2*j*_).
  The lower part of Table 5 provides the fixed effects estimates of the generalized multi-level model. Overall, the fixed effects are very similar to the effects of the aggregated logit approach. Table 6 shows the random effects of the generalized multi-level model. The variances of the random effects can be found on the main diagonal. More interestingly, we find that the random intercept correlates negatively with the random parts of the actor and partner effects (*r* = –0.71 and *r* = –0.66) indicating that in couples with high base rates of SC and DC behaviors, the occurrence of these behaviors is less strongly associated with prior behavior than for couples with lower base rates. Random components of actor and partner effects correlate positively (*r* = 0.77) indicating that in couples with larger influences from SC at *t*–1 on DC (or DC at *t*–1 on SC) we also find larger influences from DC at *t*–1 on DC (or SC at *t*–1 on SC).
  The generalized multi-level model bears the advantage that all estimates can be obtained by one single model. Furthermore, random effects and their correlations can be modeled, tested and interpreted. Additionally, the model tests whether the effects differ between the depended variables.
  However, the generalized multi-level model bears the disadvantage that the data set has to be prepared in an unusual way with one entry representing one observation of only one behavior, the two preceding behaviors of both partners and the dummy coded variable. That procedure results in twice as many entries as there are intervals minus 2 (e.g., 2 ^*^ 47 entries for one couple). Additionally, depending on the sex of the partner, actor (partner) variables represent either SC (female actor and male partner) or DC behavior (female partner and male actor). Furthermore, multi-level-models require large sample sizes for estimating the coefficient variances properly. A simulation study conducted by Maas and Hox (2005) showed that standard errors at level-2 are biased downward if level-2 sample sizes (e.g., couples) are comparably small (<50). Unbiased standard errors could be found for a sample size of 100 at level-2. Furthermore, if random effect variances are small, the estimation of model parameters can become erroneous with negative variances, for example.
- *Basic Markov Modeling* can also be used for addressing research question two. Basic Markov (or AR-1 Markov) models are based on Markov chains. A Markov chain describes a process over discrete time (Briggs and Sculpher, 1998) where the state at time *t* (e.g., showing DC) depends only on the previous state at *t*–1. This assumption is called *stationarity* (Ross, 2014) and fits perfectly to research question 2.

**Table 2:** **State-transition table for couple ID 129**.

**Prior behavior *t*–1**		**Dyadic coping (*t*)**	
**SC**	**DC**	**Yes**	**No**
Yes	Yes	23	4
Yes	No	1	1
No	Yes	3	1
No	No	3	11

*SC, stress communication; DC, dyadic coping; yes, behavior was shown; no, behavior was not shown*.

**Table 3:** **Results of logit analysis for couple ID 129**.

**Parameter**	**β**	**exp(β)**
Grand mean	0.33	1.39
DC at *t*−1 (Actor effect)	0.92	2.52^***^
SC at *t*−1 (Partner effect)	0.50	1.65^***^
Interaction effect	−0.10	0.91

*** *p < 0.001; SC, stress communication; DC, dyadic coping*.

**Table 4:** **Averaged logit parameters over all 64 couples**.

**Parameter**	**β**	**exp(β)**
Grand mean	0.25^**^	1.28^**^
DC at *t*−1 (Actor effect)	0.79^***^	2.21^***^
SC at *t*−1 (Partner effect)	0.70^***^	2.01^***^
Interaction effect	0.04	1.04

** *p < 0.01*,

*** *p < 0.001; SC, stress communication; DC, dyadic coping*.

**Table 5:** **Comparing results of aggregated logit model and MLM**.

	**Estimated** β		
	**SC**	**DC**	**DC–SC**
**AGGREGATED LOGIT MODELS**			
Mean logit	0.28^**^	0.25^**^	0.03^N^
Actor effect	1.05^***^	0.79^***^	−0.25^N^
Partner effect	0.52^***^	0.70^***^	0.27^N^
Actor^*^Parter	0.10	0.04	−0.06^N^
**MLM APPROACH**			
Mean logit	0.25^**^	0.22^*^	0.03
Actor effect	1.26^***^	0.97^***^	−0.29^***^
Partner effect	0.49^***^	0.69^***^	0.21^*^
Actor^*^Parter	0.03	0.01	−0.02

* *p < 0.05*,

** *p < 0.01*,

*** *p < 0.001, ^N^p-values not available; SC, stress communication as dependent variable; DC, dyadic coping as dependent variable; SC-DC, differences between SC and DC estimates*.

**Table 6:** **Random effect for MLM**.

	**Mean logit**	**Actor**	**Partner**
Mean logit	0.27		
Actor	−0.71	0.06	
Partner	−0.66	0.77	0.17

*Variances of level-2 residuals are on the principal diagonal; their correlations are shown in the other cells*.

Most important for the interpretation of Markov chains is the transition matrix. The cells of the transition matrix (see Table 7) depict the transition probabilities to move from a state at *t*–1 (depicted in the rows) to a particular state at *t* (depicted in the columns). Cells on the main diagonal are often interpreted as stability as they indicate the probability to remain in the particular state.

**Table 7:** **Transition probabilities for example data**.

	**->None**	**->SC**	**->DC**	**->SC+DC**
None->	0.79	0.06	0.06	0.10
SC->	0.19	0.33	0.08	0.40
DC->	0.32	0.05	0.31	0.32
SC+DC->	0.05	0.08	0.06	0.80

*None: “no stress communication and no dyadic coping”; SC, “only stress communication, but no dyadic coping”; DC, “only dyadic coping, but not stress communication”; SC+DC, “stress communication and dyadic coping”; X-> transition from X; ->X transition to X*.

In Table 7, the first cell shows that a couple showing no SC nor DC behavior at a given interval (*t*–1) will very likely (with a probability of 0.79) show no SC nor DC at the next interval (*t*). Keeping in mind that at the beginning of the interaction sequence almost all couples showed SC and DC, we can derive that once a couple attained the status of no stress reaction and no support over the curse of time, they most likely finished their coping process. The remainder of the 1st row shows that it is very unlikely that a state without SC nor DC will be followed by a state of only SC [*p*(SC|none) = 0.06], or only DC [*p*(DC|none) = 0.06]. However, a small but substantial probability of *p*(SC+DC|none) = 0.10 depicts that a state with SC and DC will occur after a state without any of the two behaviors.

The transition probabilities may also be interpreted in terms of *actor* and *partner effects*, yet the Markov model provides conditional actor and partner effects, that is the actor effect for showing SC at *t* for female partners, for example, may depend on whether the male partner displayed DC or not. Consider the case without previous display of DC, the actor effect is then calculated as the sum of the transition probabilities of the state with SC only at *t*–1 to one of the two states with display of SC (SC only and SC +DC): *p*(SC|SC) + *p*(SC+DC|SC) = 0.33 + 0.40 = 0.73 (row 2 of Table 7). In cases with additional display of DC at *t*–1, the sum of the two transition probabilities from the state with stress communication and dyadic coping at *t*–1 to the two states with stress communication at *t* reflects the conditional actor effect: *p*(SC|SC+DC) + *p*(SC+DC|SC+DC) = 0.08 + 80 = 0.88 (last row of Table 7). Male actor effect and both partner effects are calculated analogously.

Therefore, one advantage of basic Markov models is that partner*actor interactions effects can easily be interpreted in terms of probabilities. Moreover, most software packages for Markov modeling, such as the R-Packages TraMineR (Gabadinho et al., 2009) or seqHMM (Helske and Helske, 2016) can read data that is structured as sequences rendering the data handling process comparably easy. Finally, basic Markov models can be extended to hidden Markov models and to mixture Markov models, which can be used to answer research questions 3 and 4.

**Research Question 3**: Is there an underlying dyadic process, which might account for the observed behavior? Such as a latent dyadic coping process, which simultaneously affects the occurrence of DC and SC.

This question can be addressed using a hidden Markov model. In this type of Markov model, it is assumed that the (two) observed variables function as indicators of one underlying latent variable with changing status over time as presumed in the common fate model (Figure 2B). At the level of the latent variable, the process is assumed to follow a Markov chain with initial state probabilities (the probability to be in a particular state at *t* = 0; that is before the first interval) and transition probabilities.

The link between the latent process and the observed variables is defined by its emission, which is the conditional probability of observing a particular (combination of) behaviors depending on the latent state. For instances, a couple with a stressed female partner should show high rates of SC, DC and the combination of the two behaviors (i.e., high conditional probabilities to observe these behaviors if the latent state reflects the female partner's stress).

The Markov chain can be restricted to model theoretical assumptions. For example, one may assume that two latent states exist. One that correspond to a state of solving stress, and a second of having successfully coped with it. A plausible assumption would be that couples, who left a state of stress solving, will not enter it again. In terms of Markov modeling, such a state, which cannot be left once it has been entered, is called an absorbing state.

In order to demonstrate the meaning of an absorbing state, the results for the latent states model are presented in Table 8. The emissions show that state 1 can be interpreted as being in stress, because we find high probabilities of showing stress-related behaviors: *p*(SC+DC|State1 = 0.77; *p*(DC|State1) = 0.09; *p*(SC|State1) = 0.07 which sum to *p* = 0.93, the total probability of showing a stress related behavior. To the contrary, in state 2, which can be described as a state of solved or reduced stress, we find a relatively high probability of showing no stress behavior [*p*(none|State2) = 0.66].

**Table 8:** **Hidden Markov model with 2 latent states**.

**Initial states**	**State1**	**State2**		
Probabilities	1	0		
**Transitions**	**-**>**State1**	**-**>**State2**		
State1->	0.97	0.03		
State2->	0	1		
**Emissions**	**None**	**SC**	**DC**	**SC**+**DC**
State1	0.07	0.09	0.07	0.77
State2	0.65	0.09	0.12	0.14

*None: “no stress communication and no dyadic coping”; SC, “only stress communication, but no dyadic coping”; DC, “only dyadic coping, but not stress communication”; SC+DC, “stress communication and dyadic coping”; X-> transition from X; ->X transition to X; State2 was modeled as an absorbing state*.

The initial state probabilities show that all couples are initially in a state of stress and the transition matrix shows that the probabilities for leaving this state (solving the stress) is 0.03. Returning to a state of being in stress is not possible due to the model restriction. So over time, more and more couples will cope with the stress and enter state2.

Models with different numbers of latent states can be tested against each other using fit indices such as the BIC (e.g., a model with two latent states vs. a model with three latent states). Moreover, it is also possible to test if a particular hidden Markov model explains observations better than a model without any latent structure (basic Markov model). In this example, the basic Markov model (BIC: 5138) fitted better than the hidden Markov model (BIC: 5742), which can be interpreted as evidence against an underlying process.

The advantage of this model is that it allows for adapting the common fate model to sequence data. Furthermore, assumptions about the latent process can be tested via model comparisons. An additional merit is that the minimum number of sequences is one. Hence, the model can be fitted to very small sample sizes or even be used in single case analysis.

The disadvantage of the hidden Markov model is that this model is supposed for long sequences. With only few time intervals, for example three, the use of hidden Markov models is not recommended. In these cases, latent Markov models are considered more promising (Zucchini and MacDonald, 2009; see Bartolucci et al., 2015 for an implementation).

**Research Question 4**: Are there latent groups of dyads, which might account for observing different reaction patterns? Or, more specifically, can dyads be grouped according to their typical pattern of displaying SC and DC behaviors? For example, are there “fast copers” who quickly reduce the perceived stress, but also stress-prone couples (“slow copers”) who do not find their way to reduce the stress?

From the number of state-transitions, one can see that there are differences with respect to the number of observed state changes across the couples. Some are rather volatile, and others remain rather stable in their state. The focus of research question 4 is to reveal if there are several typical patterns of observed behaviors that allow for identifying groups of couples that differ in their stress treatment. Detecting unobserved groups with different response patterns in categorical data is commonly done relying on Latent Class Analysis (e.g., Collins and Wugalter, 1992; Hagenaars and McCutcheon, 2002; Asparouhov and Muthen, 2008). Hence, a first approach to answer research question 4 is to combine Latent Class Analysis and Markov modeling resulting in *mixture Markov models* (Van de Pol and Langeheine, 1990). A second approach is *sequence clustering* (Abbott, 1995).

### Mixture markov model

Mixture models or so-called latent class models assume that the population consists of several latent (unknown) subgroups. Detecting these subgroups accounts for so-called unobserved heterogeneity in the population. Dealing with sequence data, the notion of unobserved heterogeneity implies that there are different subgroups differing in their specific transition matrices. For example, if there was a group of “fast copers,” their transition probabilities into a state without SC and DC behavior would be comparably high. For “slow copers,” we would presume smaller transition probabilities to the state without SC and DC behaviors. The assignment of the couples to the latent classes is probabilistic, that is, for every couple, there are as many probabilities to belong to a particular latent class as there are classes. The couple is presumed to belong to the class with the highest assignment probability.

As a special case, a mixture Markov model with only one latent group is equivalent to the basic Markov model, hence model comparisons between models without unobserved heterogeneity and models with multiple classes are possible. For the sample data set, model comparisons did not support a Markov model with two latent classes nor with three latent classes as the associated BIC indicated worse model fit than for the basic Markov model (BIC = 5205 for two and BIC = 5292 for three latent classes compared to BIC = 5138).

However, to illustrate mixture Markov models, results of the model with two latent classes are depicted in Table 9. The latent classes contain 59 vs. 41% of the couples. The first latent class is characterized by rather stable transition probabilities to remain in the two states of showing no SC and DC behavior (*p* = 0.86) or to remain in a status with simultaneous display of SC and DC behavior (*p* = 0.84) whereas the 2nd class shows lower transition probabilities to remain in the same two states (*p* = 0.60 and *p* = 0.76, respectively). Also in the 1st class the transition probabilities to enter the state without SC and DC behavior are somewhat higher (*p* = 0.24 and *p* = 0.43 from SC behavior only and DC behavior only to the state of no SC and no DC) than in the 2nd latent class (*p* = 0.16 and *p* = 0.23).

**Table 9:** **Mixture Markov model with 2 latent groups**.

**Most probable**	**Group 1**		**Group 2**	
Group proportion	0.59		0.41	
**TRANSITIONS**				
**Group 1**	**->None**	**->SC**	**->DC**	**->SC+DC**
->None	0.86	0.03	0.05	0.07
>SC	0.24	0.23	0.09	0.44
->DC	0.43	0.06	0.34	0.17
->SC+DC	0.04	0.07	0.04	0.84
**Group 2**	**-**>**None**	**-**>**SC**	**-**>**DC**	**-**>**SC**+**DC**
->None	0.60	0.12	0.10	0.18
>SC	0.16	0.40	0.07	0.37
->DC	0.23	0.04	0.28	0.46
->SC+DC	0.07	0.09	0.09	0.76

*None: “no stress communication and no dyadic coping”; SC, “only stress communication, but no dyadic coping”; DC, “only dyadic coping, but not stress communication”; SC+DC, “stress communication and dyadic coping”; X-> transition from X; ->X transition to X*.

The advantage of this model is that it accounts for unobserved heterogeneity caused by a categorical variable. A disadvantage is that, to our knowledge, no recommendations for required sample sizes exist. However, as a simulation study by Dziak et al. (2014) showed the most basic latent class analysis needs at least 60 observations if groups are very dissimilar but several hundred for less pronounced dissimilarities.

### Sequence clustering

A second modeling approach is based upon the idea of subgrouping sequences. This modeling tradition is known as sequence analysis. However, we will refer to this approach as *sequence clustering*, because in this approach classical cluster analysis is adapted for sequence data.

Within this approach, optimal matching procedures (OM; Abbott and Tsay, 2000) can be seen as a viable alternative to mixture Markov models. Essentially, OM categorizes behavioral sequences of individuals or couples according to their similarity in a stepwise procedure: The first step is defining (dis-)similarity via an appropriate distance measure (in OM called *cost*), the second step is identifying clusters of similar sequences by applying a clustering algorithm. These clusters can be interpreted, and covariates may be included in a statistical model predicting cluster membership.

In the first step, the metric of similarity and difference between sequences is defined by Levenshtein-distances (1966): Two sequences are similar if essentially the same pattern of behavior is shown Levenshtein (1966). They differ to the extent to which some elements of one sequence have to be changed to perfectly match the other sequence (*cost*).

Consider a first case, where the sequence of couple 1 perfectly fits the sequence of couple 2 but with a shift of one interval (that is the couples exactly show the same behavioral pattern, yet the observation of couple 1 is one-time interval “behind”). For example: The first sequence is “0-1-1-1” and second sequence is “1-1-1”. In this case, the two sequences can be made identical by removing the first element in the first sequence and shifting the remainder of the sequence to the left (*deletion*), or by copying the first element of couple 1 and paste it at the beginning of the second sequence (*insertion*). Therefore, the minimal *cost* of transforming both sequences into each other is one operation.

Consider a second case with two totally identical sequences which only differ at the entry in the fourth interval. For example, the first sequence is “0-0-0-1” and the second is “0-0-0-0”. The two sequences can be made identical by *substituting* the fourth interval in the first sequence with “0” or by *substituting* the fourth interval in the second sequence with “1”. Again only one operation is needed. However, *substitution* is weighted differently than insertion or deletion, and the *cost* of this transformation would be one times a weight (*weighting*).

A higher weighting stands for more dissimilarity. The specific weighting is often derived from theoretical assumptions; however, the weighting can also be derived by applying the “TRATE”-formula of Gabadinho et al. (2009; Equation 5). That is, a basic Markov model is fitted, and the *transition probabilities* of two states, which should be substituted, are subtracted from two. Thus, if the transition between the two states is very likely, the weighting becomes smaller. The value becomes zero when each of the two states is always followed by the other. And if they never occur in consecutive order, the weight becomes two.

(5) $$\begin{array}{l} Cost\;for\;i\neq j:2-p\left(i|j\right)-p\left(j|i\right)\\ \;for\;i=j:0\\ \;i=state\;observed\;at\;time\;interval\;t\\ \;j=state\;observed\;at\;t+1 \end{array}$$

For every two observation units (couples) the minimal cost is computed for transforming their sequences into each other. The results are stored in a distances matrix with as many rows and columns as number of observations. Cells represent the minimal cost between the associated observation units. This dissimilarity matrix corresponds to other distance measures, like the Euclidian distance for example, in an ordinary cluster analysis, except that it assumes sequence data rather than metric data.

In a second step, clusters of similar sequences can be identified via a clustering algorithm. In this example, the Ward error sum of squares hierarchical clustering method (Ward, 1963) will be used because that is the default algorithm in the R-Package TraMineR (Gabadinho et al., 2009). The algorithm is commonly used (Willett, 1988), yields a unique and exact hierarchy of cluster solutions, and is comparable to most methods for identifying the number of clusters.

The algorithm treats each sequence as a single group, in the beginning. Then pairs of sequences are merged stepwise minimizing the within-group variances. The latter is determined by the squared sum of distances between each single observation and its clusters centroid. The silhouette test (Kaufman and Rousseeuw, 1990) can be used for determining which cluster solution provides the best representation of the data. The test is well-established and yields the benefit that it provides the silhouette coefficient reflecting the consistency of clusters. According to Struyf et al. (1997), a reasonable structure can be assumed if the coefficient is above 0.51.

In the application, the silhouette test resulted in a two-cluster solution with a coefficient of 0.05. Hence, additional methods should be used for validating the findings. These may include inspection of the dendrogram, scree plot, or principle component plot. All three methods lead to a two-cluster solution (see the accompanying R-script).

**Figure 6:**
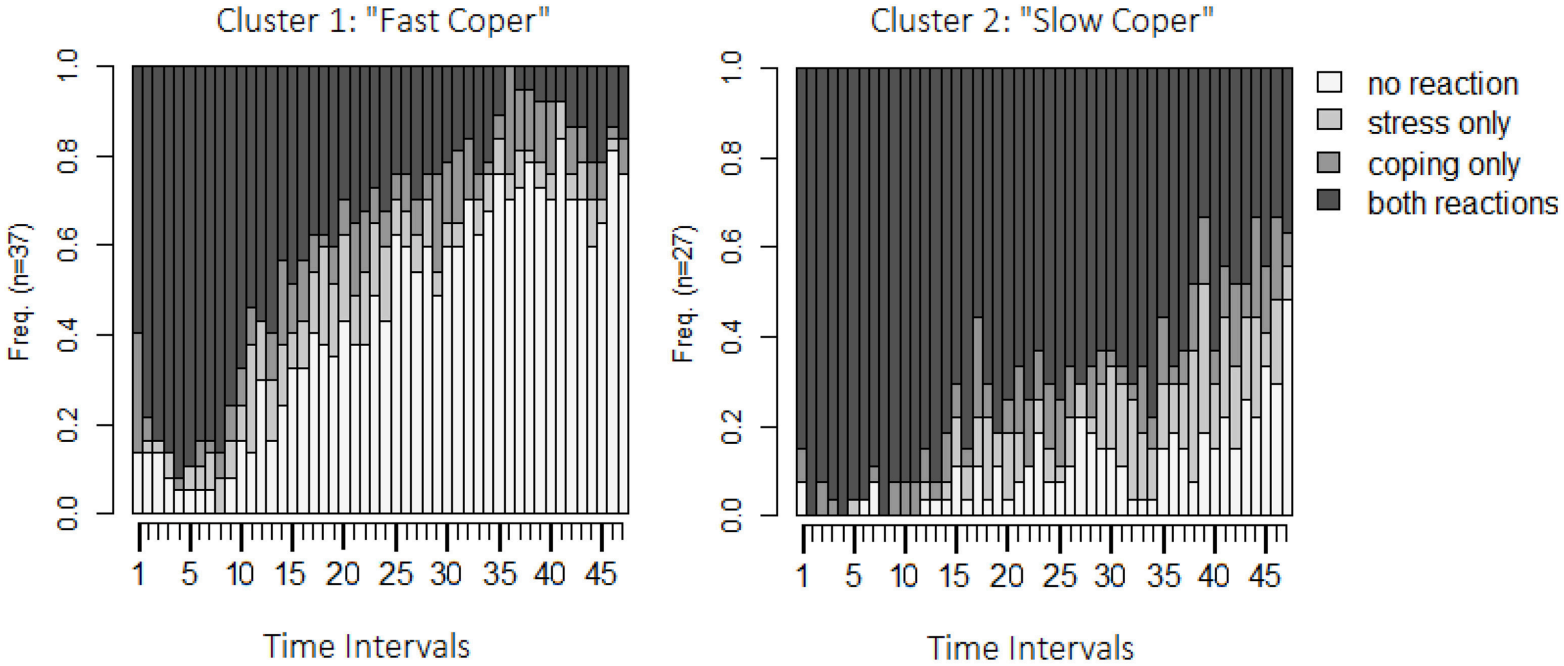
**State distribution plots both clusters identified by the OM-procedure**. Y-axis: relative frequency of shown behavior; X-axis: 48 observation intervals; SC only: only stress communication was shown but no dyadic coping; DC only: only dyadic coping was shown but no stress communication; none: neither stress communication nor DC were shown; SC+DC: stress communication and dyadic coping were shown.

The last step is to interpret or to describe the clusters. Figure 6 shows the state-distribution plots for both clusters. The obvious difference between the two clusters is that, in cluster 1, couples quickly enter states of no stress communication and no dyadic coping, whereas, in cluster 2, most of the couples remain in the state of stress communication and dyadic coping over the whole interaction sequence. Because of this, the first cluster will be referred to as the “*fast copers*” and the second as the “*slow copers.”*

Covariates can be used for further interpretations. A correlation between the cluster membership and men's self-assessed dyadic coping ability (an additional variable in the data set) reveals a weak negative correlation (*r* = −0.21), which would indicate that men in the “slow coper” cluster tend to evaluate their dyadic coping ability lower than men in the “fast coper” cluster. This association, however, is not statistically significant (*p*_two−tailed_ = 0.095).

Additional possible follow-up investigations include the application of strategies from previous sections. Table 10 provides the results of applying the aggregated logit models separately for both clusters. It shows that the actor effect is lower within the “slow coper” group, while the difference is not statistically significant, it might indicate that stress communication shown in this group is less stable or less continuously (e.g., female partners may be more often interrupted while communicating stress). The difference in partner effects is statistically significant, the partner effects is lower in the “slow coper” group. This finding indicates that the DC response for this group is less likely (or not as prompt). Furthermore, the interaction effect was not statistically significant in the overall sample. However, estimating the aggregated logit models separately for the two clusters shows that for the “fast coper” the interaction effect is negative: if stress communication is accompanied by dyadic coping the probability that stress reaction will be maintained is less than expected by the main effects. For the “slow coper” it is the opposite: if stress communication is accompanied by dyadic coping, it is more likely that it will be maintained. These findings indicate that at least two separate styles of dyadic coping might exist.

**Table 10:** **Mean log-linear parameter comparison for cluster 1 and 2 with DC as depended variable**.

**Parameter**	**Cluster 1**	**Cluster 2**	***P*^a^**
Grand mean	−0.07	0.67^***^	< 0.001
Actor effect	0.84^***^	0.73^***^	0.347
Partner effect	0.83^***^	0.52^***^	0.023
Interaction	−0.11^*^	0.24^*^	0.001

* *p < 0.05*,

*** *p < 0.001*.

a *p-value for parameter mean difference between clusters*.

Identifying the exact nature of these separate styles might be subject of further research. However, possible explanations are that the “slow copers” encourage their partners to communicate their stress by active listening, while “fast copers” tend to appease the partners. An alternative explanation might be that stressed partners of slow coping couples like to be comforted and keep their SC up so that their partners keep up their DC.

## What did we learn?

The main purpose of this article is promoting the presented analyses. Thus, a detailed substantive discussion of the findings with respect to couple research will not be provided. Instead, the overall findings will be sketched in a more general way to highlight the main interpretations of the different statistical models.

The Pearson correlation revealed a strong linear relationship between the number of observed SC and DC. Aggregated logit model and the multi-level model revealed the bidirectional effects between these two variables. The multi-level model also revealed that couples with high actor effects also tend to show higher partner effects. However, the nature of this relationship seems to be different across couples. The entropy plot shows that couples are similar at the beginning of the observation period but start to differ at interval 20 (2 min; 40 s). Sequence clustering revealed that the sample can be clustered into two groups, while the mixture Markov model did not reveal two classes. At first glance, this seems somewhat inconsistent. However, the OM-procedure assigned 70.31% of sequences to the same group as the two-class mixture model did in terms of highest probability for class membership. Thus, even though the two approaches differ with respect to the number of clusters/groups, the identified groups are comparable. Both groups are very similar in the beginning (most prominent states are “simultaneous SC and DC”) but develop differently across time. The first cluster can be characterized by a faster increase of the state without SC and DC. Additionally, this cluster (fast coper) shows a shorter duration of SC while the other (slow coper) shows much longer durations and higher rates of SC and DC states.

The aggregated logit models revealed stronger actor and partner effects for the “fast copers” than for the “slow copers,” indicating a more prompt reaction and/or simultaneous SC and DC. These effects are accompanied by a statistically significant negative interaction effect in the “fast coper” cluster, indicating that simultaneous SC and DC (or the absence of both) decreases the chance that further DC will be shown, while the opposite is true in the “slow coper” cluster. Hence, if SC is present, the DC reaction is more stable for the “fast copers.” Additionally, once SC and DC ended, the chance that it ended completely is higher in the “fast coper” cluster.

Overall the coping style of the “fast copers” seems to be more coherent (more prompt DC reactions) compared to the “slow copers.” This finding is supported by the additional correlation analysis showing that men of the “slow coper” cluster assess their DC style less good as men of the “fast coper” cluster. These findings may be used for further research questions: For example, it could be interesting to investigate if belonging to the fast vs. slow copers is more beneficial for relationship satisfaction in the long run.

## Where to find the analyses and statistical routines?

A commented R-script for reproducing all results of this paper can be downloaded at GitHub^1^. It provides a step-by-step guide through all presented analyses^2^ and instructions for installing the “DySeq”-Package (providing sample data and supplemental functions).

## Alternative approaches or choices

The *aggregated logit model, the multi-level model* and the presented *Markov models* assume stationarity. According to Helske and Helske (2016) such models can still be useful for describing data, even if stationarity cannot be assumed. However, Markov models can be extended to semi-Markov models (Yu, 2010), which do not rely on this assumption.

A disadvantage of our *multi-level adaptation* is that the model does not allow for estimating random effects for both members of a dyad separately. Kenny et al. (2006) proposed the double entry approach from Raudenbush et al. (1995) in order to obtain these estimates, however common R packages cannot handle this kind of analysis whereas the presented adaption in this contribution can be estimated by common R packages for multi-level modeling such as lme4 (Bates et al., 2015).

*Sequence clustering* is very flexible and can be configured in many different ways. Therefore, it is important to justify specifications of the statistical model or notify if the specifications were chosen arbitrarily. The substitution-cost-matrix was derived using the “TRATE”-formula from Gabadinho et al. (2009). The “TRATE”-formula is based on the transition matrix containing information about prompt changes from one state to another. Thus, it uses the same information as the aggregated logit models, and therefore, is best suited to detect subgroups with respect to prompt reactions. However, alternative methods for deriving the substitution-cost-matrix can be found in Gauthier et al. (2009).

Alternatives also exist regarding the clustering algorithm. An extensive overview of alternatives can be found in Kaufman and Rousseeuw (2009). However, one of the most appealing features of the presented Ward algorithm is that it is comparable to many other methods for determining the correct number of clusters. In some cases different tests may indicate different numbers of clusters. In these cases, additional analyses with additional variables including cluster membership as a covariate may reveal if the clusters differ from another. Meaningful differences between association patterns for these additional variables between clusters may indicate that these clusters exist. However, OM-procedures as cluster analysis are explorative in nature, and findings should be cross-validated before being generalized.

It is worth to mention that mixture Markov models can be combined with any other Markov modeling approach as well. For example, it is also possible to apply a mixture hidden Markov model (MHMM) resulting in different common fate models for latent classes. Moreover, the R-package seqHMM (Helske and Helske, 2016) provides a multi-channel approach to estimate separate emissions for each dependent variable.

## Commonly known problems and solutions in practical applications

*Dropouts* may occur meaning that observation units leave the sample before the observation period has ended. Furthermore, they may produce sequences with different lengths. However, all presented models can deal with sequences of different lengths as long as drop outs occur completely at random (MCAR). Minimal problems can occur, such as that dropouts may form their own cluster when the OM-procedure is applied.

If dropouts occur systematically, the interpretability of model results is questionable. To our knowledge, no procedures for handling data with dropouts that are not missing at random (MNAR) exist for the presented models. Methods like multiple imputation (e.g., Schafer and Graham, 2002) can be used if missingness can be considered random (MAR).

However, it is often difficult to assess if dropouts are occurring at random or systematically. Therefore, it is strongly advised to choose an experimental design that limits the risk for dropouts. In the present study, the observation took place as an 8-min long waiting condition between other parts of the experiment. The risk that a couple leaves such a condition earlier than expected is minimal. Whereas, it is very likely that dropouts occur if an observation stretches over weeks and demands multiple testing sessions.

### Zero frequencies

Some behavior may rarely be shown, or some couples may never show a certain behavior. That is especially a problem for the aggregated logit model: For all state-transition tables all cell frequencies must be larger than zero for logit models to be estimated. If some couples do never show a particular behavior, one way is excluding these couples. But, this may lead to biased estimations if zero frequencies occur systematically. An alternative is adding a constant, typically 0.5, to all cells (Everitt, 1992) to make the model estimable at the cost of a weak bias toward lower effects. It is, therefore, a conservative approach for handling zero frequencies.

The multi-level can be affected by this, too. However, the same problem is referred to as incomplete information (Field, 2013). Adding a constant to frequencies is, to our knowledge, not possible in most applications for multi-level modeling. Thus, excluding those couples is recommended.

### Low frequencies

Typically, it is advised that the predicted cell frequency in a logit model should be at least five for every cell. That is especially important for single case analysis. In this case, Hope's (1968) Monte Carlo test should be used for statistical inferences.

## Practical advices

### Observation duration

Clearly, a longer observation is always better, yet other aspects like costs, the strain on the subjects, and practical issues have to be considered. The ideal length of the observation duration depends on the subject: If the topic is an ongoing, endless process, the duration should be long enough to observe transitions between several states and long enough to generate enough time intervals for avoiding zero frequency issues. If the subject of interest is a process with a well-defined beginning and ending, the observation duration should be long enough to observe both, beginning and ending, for the vast majority of observation units. The optimal duration, in this case, may be derived from previous research, or a pilot study should be conducted.

### The length of time intervals

If interactions and changes are very fast, small intervals are needed to observe underlying patterns. Therefore, shorter intervals seem to be always better, yet this is not true for all cases. The interval length influences the estimation and meaning of stabilities and transitions. For example, if the subject of interest is the transition between jobs, and time is measured in 1-s intervals, the probability that a person will stay in her or his job the next second will be near 1. This indicates that it is very unlikely that someone will change her or his job at all. However, if time were measured in years, the probability for staying at a job might be, for example, 0.80. The interpretation changes along. The expected probability that a person will change her or his job within a year is 0.20. This example shows that it is important to consider the length of time intervals when it comes to interpretation of transition probabilities.

Consequently, this should be the central criteria for choosing an appropriate length of time intervals: The duration of time intervals should yield the most meaningful interpretation of the actual research question and can best be derived relying on prior studies or pilot studies in most cases.

The *number of intervals* depends directly on the observation duration and the length of observation intervals (observation duration divided by the length of one interval). If aggregated logit models should be applied, the number of intervals should be high enough to avoid too many zero frequency issues. Remember that every case with at least one zero frequency on their transition table will underestimate the true effects (at least if the default option for handling zero frequencies is used). If single *logit models* should be analyzed, the power depends directly on the transition probabilities and the number of intervals (in fact the time intervals become the number of observation units for this kind of analysis). Furthermore, low frequencies will occur more often if the number of intervals is low and if some transition probabilities are close to zero. Hence, one should sample as many intervals as possible.

Because plausible transition probabilities may vary strongly between several research subjects, no rules of thumbs can be given. However, the accompanying R-Package “DySeq,” provides a function (*EstFreq*) that simulates the estimated number of cells with low or zero frequencies depending on the expected mean state-transition-table. Another function (*EstTime*) computes the minimum number of time intervals that results in as many cases with low or zero frequencies as considered tolerable by the researcher. Both functions are only implemented for cases with two dichotomous coded behaviors of interest.

If the *number of coded behaviors* increases, researchers should collect much more observations. The number of cells in the transition table increases exponentially; hence, more intervals are needed. In this example, each transitions table had eight cells (2^*^2^*^2 dimensions: Number of Levels of DC times DC times Stress) containing 48 observations yielding an average absolute cell frequency of six if frequencies were distributed even over all cells. As frequencies are rarely even distributed, 48 observations seem a lower limit for the applicability of the reported models.

If, for example, DC would comprise three categories: No DC, positive, and negative DC: The transitions table would have 18 cells. Hence with 48 observations the mean frequency for even distributed data would be 2.67 (leading to low-frequency issues and very likely producing zero frequencies). If the same average cell frequency were achieved as before, 288 observation intervals would be needed.

This example illustrates that the number of cells increases dramatically the more behaviors are coded, and thereupon the number of needed intervals increases also dramatically. Therefore, coding as few different behaviors as necessary is recommended if logit models should be applied. The applicability of the OM-procedure does not depend on individual frequencies, and thus is principally applicable for any number of coded behaviors.

### The number of observation units

An ordinary power analysis of correlations can be used for research question one: According to G^*^Power 3 (Faul et al., 2007) the number of needed observations for α = 0.05 (two-tailed), 1-β = 0.80 and *r* = 0.30 is *N* = 84, for *r* = 0.50 it is *N* = 29, and for *r* = 0.80 it is *N* = 9.

For the aggregated logit models, a power analysis for *t*-testse can be used and reveals a minimum number of *N* = 199 observational units for small effects (*d* = 0.20), *N* = 34 for medium effects (*d* = 0.50), *N* = 18 for strong effects (same α and β as above). If deviations between groups should be tested (e.g., Table 10), the needed samples sizes are *N* = 788 (*d* = 0.20), *N* = 128 (*d* = 0.50), and *N* = 52 (*d* = 0.80) if groups are of equal size.

For multi-level analysis, the number of couples is more important than the number of time intervals. As mentioned before a sample size of 100 couples is recommended. Basic and hidden Markov Model can be estimated with any number of couples. However, if only one sequence should be analyzed, specialized software packages are required such as depmixS4 (Visser and Speekenbrink, 2010). For OM-distances, no power analysis or rules of thumb exist, but OM-distances have been applied successfully in studies with even relatively small sample sizes (*N* < 50; Wuerker, 1996; Blair-Loy, 1999). However, studies with many expected clusters often show bigger sample sizes, e.g., *N* = 578 for 9 clusters in Aassve et al. (2007).

Although most of the proposed models have existed for some time, they have not readily been used in psychological research on dyadic sequence data. However, we are confident that using the proposed models to investigate sequence data will broaden the range of research hypotheses in the context of social interactions and our understanding of the underlying processes. We hope to contribute to the research on sequence data by promoting these models and making their applications more accessible by providing an accompanying R-script and -package.

## Ethics statement

The study is in line with the ethical standards of the Swiss National Science Foundation (SNF). Evaluation of ethical standards were part of the approval process for the research grant. The study was promoted as a study on close relationship and stress. Participants were informed about the study protocol. Before participating in the study, they were informed that they will be exposed to stressful situations and that they will be asked to give information about their relationship quality. Participants signed an informed consent form and were informed that they could withdraw their participation at any given time without any consequence. No vunlerable populations were involved.

## Author contributions

PF: methods, model selection, statistical application, implementation in R. FN: supervisor for methods, model selection, and statistical application. NM and GB: theoretical input (e.g., providing potential research questions), contributed to discussion, sampling.

## Funding

We acknowledge support for the Article Processing Charge by the Deutsche Forschungsgemeinschaft and the Open Access Publication Fund of Bielefeld University. We gratefully acknowledge financial support from Swiss National Science Foundation awarded to Guy Bodenmann and Markus Heinrichs (SNF: 100013-115948/1 and 100014-115948).

### Conflict of interest statement

The authors declare that the research was conducted in the absence of any commercial or financial relationships that could be construed as a potential conflict of interest.

## Footnotes

^1^https://github.com/PeFox/DySeq_script

^2^Relying on the following packages “TraMineR” (Graphical analysis, state-changes, entropy, and OM-procedure; Gabadinho et al., 2009), “gmodels” and “MASS” (research question 2; Venables and Ripley, 2002; Warnes et al., 2015), “fpc” (optimal number of clusters; Henning, 2015), and “cluster” (ward algorithm; Maechler et al., 2015), “seqHMM(Markov modeling; Helske and Helske, 2016).
